# Concise Review: The Regulatory Mechanism of Lysine Acetylation in Mesenchymal Stem Cell Differentiation

**DOI:** 10.1155/2020/7618506

**Published:** 2020-01-28

**Authors:** Hong Yang, Yuexia Liu, Xuanchen Liu, Huihui Gu, Jing Zhang, Chao Sun

**Affiliations:** Key Laboratory of Animal Genetics, Breeding and Reproduction of Shaanxi Province, College of Animal Science and Technology, Northwest A&F University, Yangling, Shaanxi 712100, China

## Abstract

Nowadays, the use of MSCs has attracted considerable attention in the global science and technology field, with the self-renewal and multidirectional differentiation potential for diabetes, obesity treatment, bone repair, nerve repair, myocardial repair, and so on. Epigenetics plays an important role in the regulation of mesenchymal stem cell differentiation, which has become a research hotspot in the medical field. This review focuses on the role of lysine acetylation modification on the determination of MSC differentiation direction. During this progress, the recruitment of lysine acetyltransferases (KATs) and lysine deacetylases (KDACs) is the crux of transcriptional mechanisms in the dynamic regulation of key genes controlling MSC multidirectional differentiation.

## 1. Introduction

Mesenchymal stem cells (MSCs), a kind of adult stem cell with multidirectional differentiation potential, can differentiate into many mesodermal lineages, including adipocytes, osteoblasts, chondrocytes, muscle cells, and nerve cells [1–4]. Based on its pluripotency, MSCs represent a wide range of cell sources for the treatment of diseases such as diabetes, obesity, and autoimmune diseases and have become the focus of global scientific and technological attention [5–7]. The differentiation of MSCs is regulated by many factors [8, 9]. Epigenetics, one of the main regulatory mechanisms of MSC differentiation, plays an important role in determining cell fate [10–14]. Among them, lysine acetylation, a kind of posttranslational modification (PTM) of proteins, has been extensively studied on the regulation of transcription [15–17].

Precise control of proteins is essential to organism function. Lysine acetylation is one of the major protein modifications after translation, which has multiple effects on protein and metabolic components [18]. It can regulate the expression of genes related to multidirectional differentiation and represent the pluripotency of MSCs to a certain extent. At the same time, the degree of lysine acetylation can also affect the differentiation direction and biological function of MSCs. In this paper, the recent progress in the research of lysine acetylation modification in terms of MSC differentiation is reviewed from the above aspects.

## 2. Brief Summary of Lysine Acetylation

Lysine acetylation is a reversible process of transferring acetyl group from acetyl coenzyme A to the E-amino side chain of lysine [19]. Lysine acetylation modification is an evolutionarily conserved PTM, which exists in both prokaryotes and eukaryotes. Lysine acetylation is involved in a variety of major key cellular processes related to physiology and disease, such as gene transcription and expression, DNA damage repair, cell signal transduction, protein folding, and autophagy [20–22]. At the same time, it affects protein function through a variety of mechanisms, including protein stability, enzyme activity, subcellular localization, and other posttranslational modifications, as well as protein-protein and protein-DNA interaction, ultimately affecting cell cycle and cell differentiation [23, 24].

**Figure 1:**
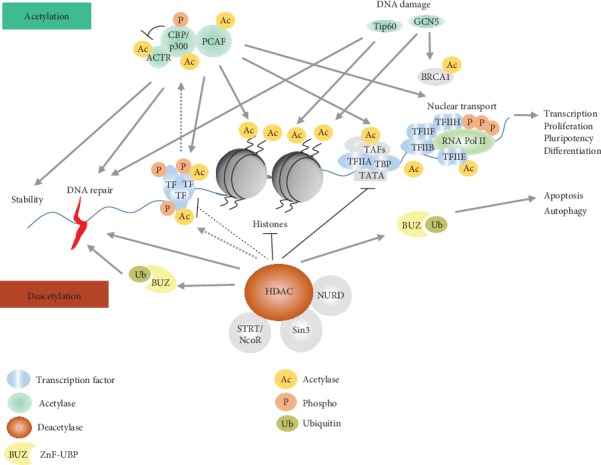
Pathway description of protein acetylation. Protein acetylation is involved in the regulation of chromatin structure and transcriptional activity. Acetylation complexes (such as CBP/p300 and PCAF) or deacetyl complexes (such as Sin3, NuRD, NcoR, and SMRT) are recruited to DNA-binding transcription factors (TFs) in response to signaling pathways. HATs induced histone hyperacetylation, which was associated with transcriptional activation, whereas HDACs induced histone deacetylation, which was associated with transcriptional repression. Many transcriptional coactivators have intrinsic acetylase activity, and transcriptional copressurization factors are associated with deacetylase activity. Histone acetylation stimulates transcription by remodeling advanced chromatin structures, attenuating histone-DNA interactions and providing binding sites for transcriptional activation complexes with proteins with containing brominated domains.

Acetylation of histones or nonhistones is mainly reversibly regulated by lysine acetyltransferase (KAT) and lysine deacetylase (KDAC), which are sometimes referred as histone acetyltransferase (HAT) and deacetylase (HDAC). KAT can relax the structure of nucleosomes, promoting the expression of transcription factors and synergy. Transcription factors can contact with DNA molecules to activate transcription of specific genes. Deacetylation is the process in which KDACs make the promoter barely access to transcriptional regulatory elements to inhibit transcription followed by gene inactivation (Figure 1).

### 2.1. Lysine Acetyltransferases (KATs) and Lysine Deacetylases (KDACs)

At present, it shows that 13 KATs have been identified in the human proteome (canonical), and most of them can be classified into three families: GCN5, p300, and MYST19 [18]. In addition, there are *α*-tubulin N-acetyltransferase 1 (TAT1/ATAT1), establishment of cohesion 1 homologue 1 (ESCO1) and ESCO2, and histone acetyltransferase 1 (HAT1/KAT1), and there is no homology. Besides TAT1, all classical KATs are mainly localized in the nucleus acetylating histones and nonhistones.

The deacetylation of proteins is catalyzed by deacetylase. At present, 18 kinds of KDACs have been found in human proteome. According to the homology of KDAC domain, it can be divided into four types: class I KDACs (HDAC1, HDAC2, HDAC3, and HDAC8), class II KDACs (class IIa: HDAC4, HDAC5, HDAC7, and HDAC9; class IIb: HDAC6 and HDAC10), class III KDACs (SirT 1-7), and class IV (including only one member, HDAC11) [25, 26]. Class I and IV KDACs are mainly distributed in the nucleus of cells, and class II KDACs are distributed in both the cytoplasm and nucleus, with exported to cytoplasm after signal activation [27]. Similarly, class III KDACs, also known as sirtuin deacetylases, are located in different cell compartments: Sirtuin 1 (SIRT1) and SIRT6 are in the nucleus, SIRT7 is in the nucleolus, SIRT2 is in the cytoplasm, and SIRT3, SIRT4, and SIRT5 are in mitochondria [28]. Moreover, class I, class II, and class IV KDACs are zinc-dependent enzymes, while class III HDACs require NAD+ as a cofactor of catalytic activity. Therefore, KDACs can also be classified into two categories: zinc-dependent HDACs and NAD^+^-dependent sirtuin deacetylases [29]. Zinc-dependent HDACs possess a highly conserved deacetylase domain, commonly referred as classical HDACs or classical KDACs (Table 1).

### 2.2. Functional Lysine Acetylation Networks

About 70% of the known acetylation sites of KATs are the targets of CBP and/or p300. Acetylation in most of the acetylated proteins is catalyzed by five KATs (CBP, p300, GCN5, PCAF, and TIP60) [27]. Similarly, for the networks regulated by KDACs, more than two-fifths of the acetylation sites are SIRT1 targets, and more than 60% are sirtuin deacetylase targets. Consistent with the location of sirtuins in cells, there are many nuclear proteins consisting of SIRT1 targets, such as transcription regulators, while SIRT3 targets are located in mitochondria, with most SIRT3 targets being involved in the regulation of mitochondrial metabolism. In contrast, KAT-regulated networks contain more transcriptional regulators, with fewer proteins being involved in metabolism.

Acetyl coenzyme A (Acetyl-CoA, ACA) is a key metabolite of cell function, including energy production in mitochondria and lipid biosynthesis in the cytoplasm. Acetylation is directly related to the level of ACA. The specific production of ACA in cells can locally affect the acetylation of proteins. For example, nuclear ACLY, ACSS2, and PDC regulate histone acetylation by locally producing ACA and thereby affecting gene transcription [30]. In yeast, the consumption of mitochondrial ACA only eliminates the acetylation of mitochondrial proteins besides nucleoproteins [31]. In mice, the loss of ACA carboxylase 1 (ACC1) and ACC2 converts ACA into malonyl coenzyme A, resulting in increased protein acetylation, which may be achieved by increasing the level of ACA [32]. By genetic and restrictive dietary methods, the researchers confirmed the correlation between the fluctuation of ACA level and the change of acetylation level, which further indicated that ACA was the limiting factor for many acetylation events [33].

### 2.3. Cellular Roles of Lysine Acetylation

Protein acetylation is associated with many cellular processes and human diseases. Line mutations in several KATs and KDACs, such as KAT6A, SMC3 (coding chromosome protein 3), and HDAC8 (coding histone deacetylase 8, SMC3 deacetylase), are related to developmental retardation, abnormalities, and mental disabilities [34, 35]. Studies have found that acetylation is also closely related to cancer, inflammation, immune, and neurometabolic diseases such as diabetes [36–38]. The fact that KATs and KDACs are deregulated in various cancers gives us a clear hint that anomalous acetylation takes place and it might be corrected by therapeutic KDAC inhibitor treatment [39, 40]. At present, many small molecule inhibitors of KDACs and KATs have been attractive therapeutic candidates [38].

Prior to the discovery of KDAC, histone deacetylase inhibitors (HDACi) also advanced protein acetylation [41, 42]. Sodium butyrate, the first compound identified to induce histone acetylation, Trichostatin A (TSA, a fungal antibiotic), valproic acid (VPA), and several other compounds were identified initially as HDACi [43–45]. Since epigenetic changes critically contributed to cancer onset and progression, HDACi were quickly recognized as promising anticancer drugs [39, 46, 47].

HDACi equally promote the acetylation of nonhistone proteins, which can determine the interactions, localization, and stability of these proteins [42]. At the cellular level, HDACi induce cell differentiation, cell cycle arrest, senescence, apoptosis, reactive oxygen species (ROS) production, and mitotic cell death. *In vivo*, HDACi can reduce the invasiveness, angiogenesis, and metastasis of tumors, thus inhibiting the development of tumors. In contrast, CBP, KAT inhibitors discovered recently, and A485, p300 inhibitor, showed antiproliferative effects on lineage-specific tumor cell lines [48]; however, KAT6A and KAT6B inhibitors induced cell senescence and inhibited mouse lymphoma growth [49].

## 3. Acetylation Modification in Differentiation of MSCs

Mesenchymal stem cells (MSCs) are pluripotent progenitor cells that have the potential to differentiate into multiple mesodermal lineages, including adipocytes, osteoblasts, and chondrocytes. During aging and osteoporosis, adipogenesis is superior to osteogenesis, which means that under these conditions, the balance of MSC differentiation is dysregulated. Numerous transcription factors are involved in the lineage selection and terminal differentiation of MSCs.

**Figure 2:**
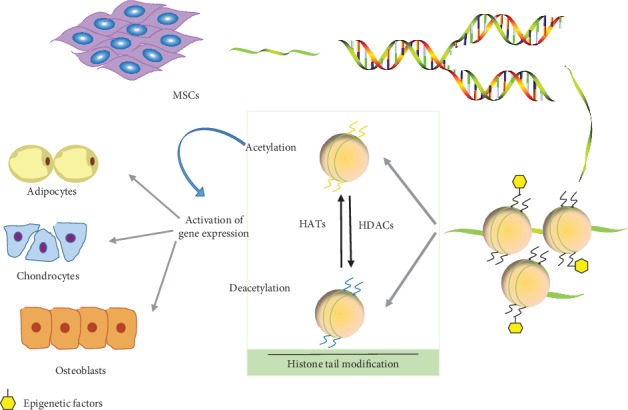
Acetylation regulation of mesenchymal stem cell differentiation along the adipocytic, osteogenic, and cartilage lineages.

Lysine acetylation regulation is involved in many cell differentiation processes. It is also one of the major regulatory mechanisms of epigenetic regulation of MSCs to adipose differentiation and osteogenic differentiation [50]. Histone acetyltransferases are involved in initiating transcription primarily by the addition of acetyl groups, which leads to DNA denaturation. HDAC can reverse the acetylation process in cells (Figure 2).

### 3.1. The Role of Acetylation Modification in Adipogenic Differentiation of MSCs (Table 2)

Lysine acetylation modification and its modified enzyme are basically involved in the epigenetic regulation of lipogenesis [51–53]. Lysine acetylation is gene-specific at adipogenic regulator genes, which play different roles in regulating transcriptional networks during adipogenesis.

Pretreatment with HDAC inhibitors VPA and sodium butyrate (NaBu) inhibited the adipogenic differentiation of human umbilical cord blood and adipose-derived mesenchymal stem cells [54]; HDAC inhibitors TSA and suberoylanilide hydroxamic acid (SAHA) could inhibit the adipogenic differentiation of human preadipocytes [55]; the differentiation of fat cells could be promoted with *hdac3* knockout or the expression of *hdac1* interfered with siRNA [56–58]. In addition, high expression level of HDAC5 and HDAC6 is required for adequate adipocyte function [59]. HDAC9 has been demonstrated to repress adipogenesis. In the case of a chronic high-fat diet, proper adipogenic differentiation is impaired, and the expression of a negative regulator of adipogenic HDAC9 is increased. Ablation of HDAC9 in mice can prevent adverse health effects of chronic high-fat diets, including weight gain, impaired glucose tolerance, and insulin insensitivity [12, 60, 61]. Therefore, HDAC inhibitions hold great promise for clinical targeting of obesity-related diseases.

Multiple transcription factors are involved in the lineage selection and terminal differentiation of MSCs. Peroxisome proliferator-activated receptor *γ* (PPAR*γ*), CCAAT/enhancer-binding proteins (C/EBPs), adipocyte assay and differentiation-dependent factor 1/sterol response element-binding protein 1c (ADD1/SREBP1c) are key regulators of mammalian adipocyte differentiation and also participate in the spectrum selection and terminal differentiation of MSCs [62, 63]. Studies have shown that the activation of PPAR*γ* and C/EBP*α* is accomplished by the interaction of transcription factors, coactivators, and coinhibitors. Among them, lysine acetylation and deacetylation play an important role by genetic regulation. Zhang et al. (2012) detected the distribution patterns of acetylation modification of five key adipose formation regulatory genes, Pref-1, C/EBP*β*, C/EBP*α*, PPAR*γ*2, and aP2, during the adipogenesis of C3H 10T1/2 mouse mesenchymal stem cells (MSCs) and 3T3-L1 preadipocytes, in order to determine the role of acetylation modification of lysine and its “division of labor” in adipocyte differentiation. The results showed that the detected lysine acetylation modification was globally stable throughout the adipogenesis process but showed a unique and highly dynamic distribution pattern for specific genes. For example, PPAR*α* 2 and aP2 genes in MSCs showed increased histone acetylation in the tails of H3 and H4 during adipogenesis, and increased histone acetylation levels activate the transcription of PPAR*α* 2 and aP2 genes [64]. In addition to directly binding to the lipid modifier genes, histone-modifying enzymes also modulate adipogenesis by interacting with adipogenic regulators. For example, SIRT1, a representative member of the mammalian sirtuin family, attenuates adipogenesis by binding to the major regulatory factor PPAR*γ* and inhibiting its target genes when food is restricted [65–71]. Conversely, decreased Sirt1 leads to an increase in PPAR*γ* acetylation, thereby increasing C/EBP*α* expression and promoting the development of lipogenesis [72]. In addition, KATs p300/CBP and Tip60 promote the activation of adipogenic-related genes by directly interacting with PPAR*γ*, thereby enhancing its transcriptional processes [73, 74]. Moreover, HDAC1 has been shown to be associated with regulatory elements of BAT-specific genes, leading to a decrease in the degree of histone H3K27 acetylation and thus to transcriptional inhibition [75]. And HDAC3 is also involved in the regulation of BAT gene expression and thermogenesis in mice [76–78]. Interestingly, unlike HDAC1- and HDAC3-mediated regulation, HDAC9-mediated regulation of adipogenic gene expression is not dependent on its deacetylase domain, but instead on the control of amino-terminal cofactor interaction module of the protein [76].

### 3.2. The Role of Acetylation Modification in Osteogenic Differentiation of MSCs (Table 3)

The degree of histone acetylation of related regulatory genes may reflect the maintenance and differentiation status of MSCs [79]. The acetylation of H3K9 and H3K14 (H3K9ac, H3K14ac) is a marker of gene activation [80]. During the osteogenic differentiation of bone marrow mesenchymal stem cells (BMMSCs), the expression of osteogenesis-related genes RUNX2 and alkaline phosphatase (ALP) gradually increased, while the expression of stem factors Oct4 and Sox2 related to stem cell self-renewal decreased significantly, and the variation was closely related to H3K9ac and H3K14ac [81].

In the current study, class I HDAC1, HDAC2, HDAC3, and HDAC8 and class III SIRT1 and SIRT3 played an important role in the differentiation direction of BMMSCs [82–89]. In the myocardial microenvironment, BMMSCs can differentiate into cardiomyocytes. During this process, the expression of HDAC1 is significantly decreased. At the same time, knockdown of HDAC1 can promote the direct differentiation of BMMSCs into cardiomyocytes [90]. HDAC8 reduces the osteogenic differentiation of rat BMMSCs by inhibiting the acetylation of H3K9 and the activity of RUNX2 [83].

SIRT1 can directly regulate the factor Sox2 to maintain the self-renewal and pluripotency of BMMSCs. The decrease of its activity reduces the expression of Sox2, which leads to the degradation of self-renewal and differentiation ability of BMMSCs. The activated SIRT1 can dose-dependently promote the ability of BMMSCs to clone and differentiate into osteogenic adipogenic differentiation [91]. Similarly, SIRT1 can regulate the transcription of genes involved in BMMSC differentiation by deacetylating *β*-catenin to accumulate in the nucleus [92]. In addition, SIRT1 promotes the cartilage differentiation process of BMMSCs by activating the deacetylation of Sox9 and NF-*κ*B [93].

Histone deacetylase inhibitors have a strong influence on the differentiation of BMMSCs. Treatment of BMMSCs with histone deacetylase inhibitors VPA and NaBu increased histone H3 and H4 acetylation levels and significantly promoted liver-specific gene expression, suggesting that the agent promotes the differentiation of BMMSCs into the liver by inhibiting deacetylase [94]. At the same time, NaBu inhibited the expression of HDAC2 in rat BMMSCs and its recruitment on smooth muscle-specific genes could further induce high levels of H3K9ac and H4ac, which promoted the expression of smooth muscle-specific genes and induced BMMSCs to differentiate into smooth muscle [95]. Another histone deacetylase inhibitor, TSA, significantly inhibits the decreasing of Oct4, Sox2, and Nanog to stabilize the expression of pluripotency genes in BMMSCs [96]. Other studies have found that TSA treatment increases the level of acetylation of histone H3 and inhibits adipogenic differentiation of BMMSCs [97].

In addition, Tan et al. studied the H3K9ac modification of the gene promoter region of hBMMSCs at the genome-wide level. The results showed that the modification of H3K9 in the promoter region of hBMMSCs correlated well with mRNA expression. Functional analysis showed self-renewal in hBMMSCs. Multiple key intracellular signal transduction pathways can be regulated by H3K9 modification [98]. In the process of osteogenic differentiation of hBMMSCs, the overall enrichment of H3K9ac in the promoter region of the gene is gradually reduced [99]. *In vitro*, chondrogenic differentiation of hBMMSCs significantly increased the level of chromatin marker H3K9ac at the promoter and 5′ end regions of the gene [100]. These results suggested that gene activation and silencing affected by H3K9ac may be critical for self-renewal, pluripotency maintenance, and osteogenic differentiation of MSCs.

### 3.3. The Role of Acetylation Modification in the Differentiation of MSCs into Chondrocytes

In cartilage tissue regeneration medicine, lysine acetylation is involved in the regulation of chondrocyte differentiation and terminal differentiation of mesenchymal stem cells. Cartilage tissue is a vascularless tissue composed of chondrocyte and extracellular matrix. Hence, cartilage tissue has limited repair ability [101]. Mesenchymal stem cells (MSCs) are promising alternative sources of chondrocytes because of their long-term self-renewal and multidirectional differentiation potential. The differentiation of mesenchymal stem cells into chondrocytes is essentially a process of chondrocyte-specific phenotype gene expression in the mesenchymal stem cell genome. Various signaling pathways including transforming growth factor-*β* 1 (TGF-*β* 1)/SMAD pathway and Wnt/*β*-catenin pathway have been proved to be related to this process [102–107]. Lysine acetylation plays an important role in regulating the expression of cartilage-specific genes [108–111]. Histone modification controls expression of key genes in cartilage formation by altering the spatial structure of chromatin, ultimately regulating the process of chondrogenesis of stem cells.

Histone modification plays an important role in regulating the early chondrogenic differentiation of mesenchymal stem cells [112]. Coactivator P300 has histone acetylase activity, which can directly mediate histone acetylation of Sox9 and activate Sox9 for cartilage formation. P300 can also interact with cyclic adenosine phosphate effector binding protein (CREB) to form coactivator and Sox9 to enhance the expression of chondrocyte-specific phenotype gene Col2al. Thus, the histone acetylation modification associated with P300 can regulate the expression of chondrocyte-specific genes Sox9 and Col2al [113–115]. HDAC1 can not only directly bind to the promoter region of *β*-catenin to inhibit the expression of *β-catenin* gene but also degrade *β*-catenin through the interaction between the domain of deacetylase and the deacetylated *β*-catenin protein, which leads to the downregulation of the classical Wnt/*β*-catenin signaling pathway and promotion of the cartilage differentiation process of mouse mesenchymal stem cells induced by TGF-*β* 1 [116]. HDAC4 can also promote the chondrogenesis of porcine synovial-derived mesenchymal stem cells (SDSCs) induced by TGF-*β* 1; meanwhile, HDAC4 can inhibit the expression of X hypertrophic phenotype X (SDSCs) [117].

Cartilage damage is usually accompanied by the occurrence of bone lesions. Multiple local factors are involved in regulating the physiological remodeling of cartilage, and the loss of balance of these factors may result in higher cartilage catabolism. Molecules of the Wnt pathway have become key regulators of bone and cartilage. Activation of Wnt/*β*-catenin induces an imbalance in cartilage homeostasis [102, 118]. *In vitro* chondrogenesis experiments using C3H10T1/2 cells showed that mRNA and protein levels of *β*-catenin were inhibited during chondrogenesis, while expression levels of HDAC1 was elevated. The opposite expression pattern between *β*-catenin and HDAC1 suggests that there may be novel regulatory mechanisms involved in cartilage formation between these two factors [116].

## 4. Conclusion and Outlook

Lysine acetylation regulation is involved in many cell development and differentiation processes.

In the complex and delicate internal environment of organisms, epigenetic regulation often does not work in a single way. Different histone modifications can interact with each other and play a synergistic role. Histone modification can also be coupled with DNA methylation to produce complex epigenetic effects. The network regulation pattern of epigenetic modification is also involved in the fine regulation of adipogenic differentiation of MSCs. The balance between osteogenic and adipogenic differentiation of MSCs is regulated by DNA methylation and histone acetylation in the promoter region of C/EBP*α* [119, 120]. At the end of osteogenic differentiation, the hypermethylation of C/EBP*α* promoter region prevents binding of PPAR*γ* with HDAC1 binding to this region further, reducing histone acetylation levels, and PPAR*γ* establishes DNA methylation in the promoter region of C/EBP*α* and the bridge of histone acetylation [120]. Histone modification factor YY1 and transcriptional coactivator p300 can alter the expression of chondrocyte-specific gene ChM-I in BMMSCs by regulating the level of histone acetylation, inhibiting YY1 and increasing p300 and hypomethylation of the promoter region. The expression of basic transcription factor Specificity 3 (Sp3) maintains the expression of ChM-I but does not function in the same way in hypermethylated cells, suggesting that there is synergistic negative regulation of ChM-1 by histidine deacetylation and methylation during BMMSC cartilage differentiation [121].

RUNX2 was upregulated in BMMSCs during osteogenic differentiation, and both transcriptional activation-related H3K9ac and H3K4me3 modification levels and recruitment in the RUNX2 promoter region were elevated, while H3K9me3 modification levels associated with transcriptional repression were observed in the RUNX2 promoter region. The recruitment was reduced, and the degree of DNA methylation in the RUNX2 promoter region was reduced [97]. These findings suggested that different epigenetic modifications can synergistically regulate the differentiation process of BMMSCs.

Epigenetic modulators affect the function of adult tissue stem cells primarily by modulating the function of tissue-specific master regulators. However, for us, it is still far away to understand the specific role of individual epigenetic factors; more importantly, their combined activity in adult stem cells and their communication is unclear. We face many technical challenges, such as *in vivo* generation of models to specifically study stem cells and their molecular regulatory mechanisms of adult origin, as well as the lack of stem cell-specific inducible targeting strains and conventional methods for epigenetic analysis from very small amounts and powerful calculation methods to understand the large amount of data generated.

In recent years, a variety of epigenetic modifications have been found to participate in the differentiation of MSCs. Based on these modifications, drugs have been developed to effectively regulate these modifications, providing precise differentiation conditions for MSCs and enabling MSCs to differentiate in a controllable and predictable direction. At present, some small-molecule drugs that can regulate stem cell differentiation and proliferation are in the stage of detection and development, which can participate in various aspects of regulatory programming and development signaling pathways [122]. These results also have promising value for the study of differentiation mechanism and clinical application of mesenchymal stem cells derived from the bone marrow, fat, and umbilical cord blood.

In conclusion, the regulation of lysine acetylation plays an important role in the process of MSC adipogenesis and differentiation, but the specific mechanism is not yet fully understood, and a new regulatory modification network needs to be found. Further research in this field will provide clues for the fate of MSC differentiation and will have broad application prospects in clinical tissue engineering and cell therapy.

## Figures and Tables

**Table 1:** The classification of KATs/KDACs.

Enzyme	Family		Abbreviations	Subcellular localization
KATs	GCN5		GCN5 (KAT2A), PCAF (KAT2B)	Nucleus
	P300		CBP (KAT3A), P300 (KAT3B)	Nucleus
	MYST		Tip60 (KAT5), MOZ (KAT6A) MORF (KAT6B), HBO1 (KAT7), MOF (KAT8)	Nucleus
	Other		ESCO1, ESCO2, HAT1	Nucleus
			TAT1	Cytoplasm

KDACs	Class I		HDAC1, HDAC2, HDAC3, HDAC8	Nucleus
	Class II	Class IIa	HDAC4, HDAC5, HDAC7, HDAC9	Nucleus
		Class IIb	HDAC6, HDAC10	Cytoplasm
	Class IV		HDAC11	Nucleus
	Class III (SIRT)		SIRT1, SIRT6	Nucleus
			SIRT2	Cytoplasm
			SIRT3, SIRT4, SIRT5	Mitochondria
			SIRT7	Nucleolus

**Table 2:** Acetylation regulation of mesenchymal stem cell differentiation into adipose cells.

Adipogenesis				
Epigenetic mark/enzymatic function	Specific chromatin modifier	Targeted stem cell population	Differentiation	Refs.
Deacetylation				
	HDAC3	3 T3-L1	Attenuates adipogenesis by binding to the master regulator PPAR*γ* and attenuating PPAR*γ*'s capacity to drive gene expression	[56, 58]
	Sirt1	Mice bone marrow	Attenuates adipogenesis by binding to the master regulator PPAR*γ* and repressing its target genes	[62, 63, 65–72]
			$\text{Sirt}1↓\left\{\begin{array}{c} \text{Increase of acetylated PPAR}\gamma \to \text{increased C}/\text{EBP}\alpha \text{ expression}\\ \begin{array}{c} \to \text{promoted adipogenesis}\\ \text{increase of acetylated Sox}9\to \text{reduction of collagen }2\alpha 1 \end{array}\\ \to \text{impaired chondrogenic differentiation} \end{array}\right.$	[66]

**Table 3:** Acetylation regulation of mesenchymal stem cell differentiation into osteoblasts.

Osteoblastogenesis				
Epigenetic mark/enzymatic function	Specific chromatin modifier	Nonhistone substrates or interacting proteins	Differentiation function	Ref.
Deacetylation				
	HDAC1, HDAC2		↓	[85–87, 89]
	HDAC3, HDAC7	Interaction with RUNX2	↓	[84, 88]
	HDAC4, HDAC5	RUNX2 deacetylation; interaction with SMAD3	↓	[68, 69]
	HDAC8	H3K9ac	↓	[83]
	SIRT1	Beta-catenin deacetylation	↑	[90–92]
	SIRT3	SOD deacetylation	↑	[89]

↑: promotion of differentiation; ↓: suppression of differentiation.

## References

[1] Mochizuki T., Muneta T., Sakaguchi Y. (2006). Higher chondrogenic potential of fibrous synovium- and adipose synovium-derived cells compared with subcutaneous fat-derived cells: distinguishing properties of mesenchymal stem cells in humans. *Arthritis & Rheumatism*.

[2] Rodriguez A.-M., Elabd C., Delteil F. (2004). Adipocyte differentiation of multipotent cells established from human adipose tissue. *Biochemical and Biophysical Research Communications*.

[3] Rangappa S., Fen C., Lee E. H., Bongso A., Sim E. K. (2003). Transformation of adult mesenchymal stem cells isolated from the fatty tissue into cardiomyocytes. *Annals of Thoracic Surgery*.

[4] Jurgens W. J. F. M., Kroeze R. J., Zandieh-Doulabi B. (2013). One-step surgical procedure for the treatment of osteochondral defects with adipose-derived stem cells in a caprine knee defect: a pilot study. *Bioresearch Open Access*.

[5] Lai P., Weng J., Guo L., Chen X., Du X. (2019). Novel insights into MSC-EVs therapy for immune diseases. *Biomarker Research*.

[6] Marrazzo P., Crupi A. N., Alviano F., Teodori L., Bonsi L. (2019). Exploring the roles of MSCs in infections: focus on bacterial diseases. *Journal of Molecular Medicine*.

[7] Shi M., Yuan Y., Liu J. (2018). MSCs protect endothelial cells from inflammatory injury partially by secreting STC1. *International Immunopharmacology*.

[8] James A. W. (2013). Review of signaling pathways governing MSC osteogenic and adipogenic differentiation. *Scientifica*.

[9] Zhang Y., Marsboom G., Toth P. T., Rehman J. (2013). Mitochondrial respiration regulates adipogenic differentiation of human mesenchymal stem cells. *PLoS One*.

[10] Ye L., Fan Z., Yu B. (2012). Histone demethylases KDM4B and KDM6B promotes osteogenic differentiation of human MSCs. *Cell Stem Cell*.

[11] Hemming S., Cakouros D., Isenmann S. (2014). EZH2 and KDM6A act as an epigenetic switch to regulate mesenchymal stem cell lineage specification. *Stem Cells*.

[12] Chatterjee T. K., Basford J. E., Knoll E. (2014). HDAC9 knockout mice are protected from adipose tissue dysfunction and systemic metabolic disease during high-fat feeding. *Diabetes*.

[13] Laine S. K., Alm J. J., Virtanen S. P., Aro H. T., Laitala-Leinonen T. K. (2012). MicroRNAs miR-96, miR-124, and miR-199a regulate gene expression in human bone marrow-derived mesenchymal stem cells. *Journal of Cellular Biochemistry*.

[14] Bork S., Horn P., Castoldi M., Hellwig I., Ho A. D., Wagner W. (2011). Adipogenic differentiation of human mesenchymal stromal cells is down-regulated by microRNA-369-5p and up-regulated by microRNA-371. *Journal of Cellular Physiology*.

[15] Chen H., Liu X., Chen H. (2014). Role of SIRT1 and AMPK in mesenchymal stem cells differentiation. *Ageing Research Reviews*.

[16] Yin N., Lu R., Lin J., Zhi S., Tian J., Zhu J. (2014). Islet-1 promotes the cardiac-specific differentiation of mesenchymal stem cells through the regulation of histone acetylation. *International Journal of Molecular Medicine*.

[17] Wang H., Hu Z., Wu J. (2019). Sirt1 promotes osteogenic differentiation and increases alveolar bone mass via bmi1 activation in mice. *Journal of Bone and Mineral Research*.

[18] Drazic A., Myklebust L. M., Ree R., Arnesen T. (2016). The world of protein acetylation. *Biochimica et Biophysica Acta*.

[19] Allfrey V. G., Faulkner R., Mirsky A. E. (1964). Acetylation and methylation of histones and their possible role in the regulation of RNA synthesis. *Proceedings of the National Academy of Sciences of the United States of America*.

[20] Alomer R. M., da Silva E. M. L., Chen J. (2017). Esco1 and Esco2 regulate distinct cohesin functions during cell cycle progression. *Proceedings of the National Academy of Sciences of the United States of America*.

[21] Guo X., Bai Y., Zhao M. (2018). Acetylation of 53BP1 dictates the DNA double strand break repair pathway. *Nucleic Acids Research*.

[22] Lin S. Y., Li T. Y., Liu Q. (2012). GSK3-TIP60-ULK1 signaling pathway links growth factor deprivation to autophagy. *Science*.

[23] Mujtaba S., He Y., Zeng L. (2002). Structural basis of lysine-acetylated HIV-1 Tat recognition by PCAF bromodomain. *Molecular Cell*.

[24] Li T., Diner B. A., Chen J., Cristea I. M. (2012). Acetylation modulates cellular distribution and DNA sensing ability of interferon-inducible protein IFI16. *Proceedings of the National Academy of Sciences of the United States of America*.

[25] Haberland M., Montgomery R. L., Olson E. N. (2009). The many roles of histone deacetylases in development and physiology: implications for disease and therapy. *Nature Reviews Genetics*.

[26] Gregoretti I., Lee Y. M., Goodson H. V. (2004). Molecular evolution of the histone deacetylase family: functional implications of phylogenetic analysis. *Journal of Molecular Biology*.

[27] Narita T., Weinert B. T., Choudhary C. (2019). Functions and mechanisms of non-histone protein acetylation. *Nature Reviews Molecular Cell Biology*.

[28] Houtkooper R. H., Pirinen E., Auwerx J. (2012). Sirtuins as regulators of metabolism and healthspan. *Nature Reviews Molecular Cell Biology*.

[29] Van Dyke M. W. (2014). Lysine deacetylase (KDAC) regulatory pathways: an alternative approach to selective modulation. *ChemMedChem*.

[30] Sivanand S., Viney I., Wellen K. E. (2018). Spatiotemporal control of acetyl-CoA metabolism in chromatin regulation. *Trends in Biochemical Sciences*.

[31] Weinert B. T., Iesmantavicius V., Moustafa T. (2014). Acetylation dynamics and stoichiometry in Saccharomyces cerevisiae. *Molecular Systems Biology*.

[32] Chow J. D. Y., Lawrence R. T., Healy M. E. (2014). Genetic inhibition of hepatic acetyl-CoA carboxylase activity increases liver fat and alters global protein acetylation. *Molecular Metabolism*.

[33] Carrico C., Meyer J. G., He W., Gibson B. W., Verdin E. (2018). The mitochondrial acylome emerges: proteomics, regulation by sirtuins, and metabolic and disease implications. *Cell Metabolism*.

[34] Mattioli F., Schaefer E., Magee A. (2017). Mutations in histone acetylase modifier BRPF1 cause an autosomal-dominant form of intellectual disability with associated ptosis. *American Journal of Human Genetics*.

[35] Yan K., Rousseau J., Littlejohn R. O. (2017). Mutations in the chromatin regulator gene BRPF1 cause syndromic intellectual disability and deficient histone acetylation. *American Journal of Human Genetics*.

[36] Pasqualucci L., Dominguez-Sola D., Chiarenza A. (2011). Inactivating mutations of acetyltransferase genes in B-cell lymphoma. *Nature*.

[37] Mullighan C. G., Zhang J., Kasper L. H. (2011). *CREBBP* mutations in relapsed acute lymphoblastic leukaemia. *Nature*.

[38] Falkenberg K. J., Johnstone R. W. (2014). Histone deacetylases and their inhibitors in cancer, neurological diseases and immune disorders. *Nature Reviews Drug Discovery*.

[39] Bolden J. E., Peart M. J., Johnstone R. W. (2006). Anticancer activities of histone deacetylase inhibitors. *Nature Reviews Drug Discovery*.

[40] Das C., Kundu T. (2005). Transcriptional regulation by the acetylation of nonhistone proteins in humans -- a new target for therapeutics. *IUBMB Life (International Union of Biochemistry and Molecular Biology: Life)*.

[41] Caron C., Boyault C., Khochbin S. (2005). Regulatory cross-talk between lysine acetylation and ubiquitination: role in the control of protein stability. *BioEssays*.

[42] Glozak M. A., Sengupta N., Zhang X., Seto E. (2005). Acetylation and deacetylation of non-histone proteins. *Gene*.

[43] Riggs M. G., Whittaker R. G., Neumann J. R., Ingram V. M. (1977). n -Butyrate causes histone modification in HeLa and Friend erythroleukaemia cells. *Nature*.

[44] Yoshida M. (2007). Potent and specific inhibition of mammalian histone deacetylase both in vivo and in vitro by trichostatin A. *Tanpakushitsu Kakusan Koso Protein Nucleic Acid Enzyme*.

[45] Göttlicher M., Minucci S., Zhu P. (2001). Valproic acid defines a novel class of HDAC inhibitors inducing differentiation of transformed cells. *EMBO Journal*.

[46] Warrell R. P., He L. Z., Richon V., Calleja E., Pandolfi P. P. (1998). Therapeutic targeting of transcription in acute promyelocytic leukemia by use of an inhibitor of histone deacetylase. *Journal of the National Cancer Institute*.

[47] Yoo C. B., Jones P. A. (2006). Epigenetic therapy of cancer: past, present and future. *Nature Reviews Drug Discovery*.

[48] Lasko L. M., Jakob C. G., Edalji R. P. (2017). Discovery of a selective catalytic p300/CBP inhibitor that targets lineage-specific tumours. *Nature*.

[49] Baell J. B., Leaver D. J., Hermans S. J. (2018). Inhibitors of histone acetyltransferases KAT6A/B induce senescence and arrest tumour growth. *Nature*.

[50] Dudakovic A., Camilleri E. T., Lewallen E. A. (2015). Histone deacetylase inhibition destabilizes the multi-potent state of uncommitted adipose-derived mesenchymal stromal cells. *Journal of Cellular Physiology*.

[51] Lin H. P., Cheng Z. L., He R. Y. (2016). Destabilization of fatty acid synthase by acetylation inhibits de novo lipogenesis and tumor cell growth. *Cancer Research*.

[52] Kim M. K., Kim E. J., Kim J. E., Lee D. H., Chung J. H. (2018). Anacardic acid reduces lipogenesis in human differentiated adipocytes _via_ inhibition of histone acetylation. *Journal of Dermatological Science*.

[53] Wan W., You Z., Xu Y. (2017). mTORC1 phosphorylates acetyltransferase p300 to regulate autophagy and lipogenesis. *Molecular Cell*.

[54] Lee S., Park J.-R., Seo M.-S. (2009). Histone deacetylase inhibitors decrease proliferation potential and multilineage differentiation capability of human mesenchymal stem cells. *Cell Proliferation*.

[55] Catalioto R.-M., Maggi C. A., Giuliani S. (2009). Chemically distinct HDAC inhibitors prevent adipose conversion of subcutaneous human white preadipocytes at an early stage of the differentiation program. *Experimental Cell Research*.

[56] Knutson S. K., Chyla B. J., Amann J. M., Bhaskara S., Huppert S. S., Hiebert S. W. (2008). Liver-specific deletion of histone deacetylase 3 disrupts metabolic transcriptional networks. *The EMBO Journal*.

[57] Lee J.-H., Kim K.-A., Kwon K.-B. (2007). Diallyl disulfide accelerates adipogenesis in 3T3-L1 cells. *International Journal of Molecular Medicine*.

[58] Fajas L., Egler V., Reiter R. (2002). The retinoblastoma-histone deacetylase 3 complex inhibits PPARgamma and adipocyte differentiation. *Developmental Cell*.

[59] Catalano M. G., Poli R., Pugliese M., Fortunati N., Boccuzzi G. (2007). Valproic acid enhances tubulin acetylation and apoptotic activity of paclitaxel on anaplastic thyroid cancer cell lines. *Endocrine-Related Cancer*.

[60] Chatterjee T. K., Basford J. E., Yiew K. H., Stepp D. W., Hui D. Y., Weintraub N. L. (2014). Role of histone deacetylase 9 in regulating adipogenic differentiation and high fat diet-induced metabolic disease. *Adipocyte*.

[61] Chatterjee T. K., Idelman G., Blanco V. (2011). Histone deacetylase 9 is a negative regulator of adipogenic differentiation. *Journal of Biological Chemistry*.

[62] Farmer S. R. (2006). Transcriptional control of adipocyte formation. *Cell Metabolism*.

[63] Li Q., Peng H., Fan H. (2016). The LIM protein Ajuba promotes adipogenesis by enhancing PPAR *γ* and p300/CBP interaction. *Cell Death & Differentiation*.

[64] Zhang Q., Ramlee M. K., Brunmeir R., Villanueva C. J., Halperin D., Xu F. (2012). Dynamic and distinct histone modifications modulate the expression of key adipogenesis regulatory genes. *Cell Cycle*.

[65] Picard F., Kurtev M., Chung N. (2004). Sirt1 promotes fat mobilization in white adipocytes by repressing PPAR-*γ*. *Nature*.

[66] Lin C.-H., Li N.-T., Cheng H.-S., Yen M.-L. (2017). Oxidative stress induces imbalance of adipogenic/osteoblastic lineage commitment in mesenchymal stem cells through decreasing SIRT1 functions. *Journal of Cellular and Molecular Medicine*.

[67] Jang M. J., Park U.-H., Kim J. W., Choi H., Um S.-J., Kim E.-J. (2017). CACUL1 reciprocally regulates SIRT1 and LSD1 to repress PPAR*γ* and inhibit adipogenesis. *Cell Death & Disease*.

[68] Li Y., He X., Li Y. (2011). Nicotinamide phosphoribosyltransferase (Nampt) affects the lineage fate determination of mesenchymal stem cells: a possible cause for reduced osteogenesis and increased adipogenesis in older individuals. *Journal of Bone and Mineral Research*.

[69] Peltz L., Gomez J., Marquez M. (2012). Resveratrol exerts dosage and duration dependent effect on human mesenchymal stem cell development. *PLoS One*.

[70] Puri N., Sodhi K., Haarstad M. (2012). Heme induced oxidative stress attenuates sirtuin1 and enhances adipogenesis in mesenchymal stem cells and mouse pre-adipocytes. *Journal of Cellular Biochemistry*.

[71] Bäckesjö C.-M., Li Y., Lindgren U., Haldosén L.-A. (2009). Activation of Sirt1 decreases adipocyte formation during osteoblast differentiation of mesenchymal stem cells. *Cells, Tissues, Organs*.

[72] Qu P., Wang L., Min Y., McKennett L., Keller J. R., Lin P. C. (2016). Vav1 regulates mesenchymal stem cell differentiation decision between adipocyte and chondrocyte via Sirt1. *Stem Cells*.

[73] Gelman L., Zhou G., Fajas L., Raspé E., Fruchart J. C., Auwerx J. (1999). p300 interacts with the N- and C-terminal part of PPARgamma2 in a ligand-independent and -dependent manner, respectively. *Journal of Biological Chemistry*.

[74] van Beekum O., Brenkman A. B., Grøntved L. (2008). The adipogenic acetyltransferase Tip60 targets activation function 1 of peroxisome proliferator-activated receptor gamma. *Endocrinology*.

[75] Li F., Wu R., Cui X. (2016). Histone deacetylase 1 (HDAC1) negatively regulates thermogenic program in brown adipocytes via coordinated regulation of histone H3 lysine 27 (H3K27) deacetylation and methylation. *Journal of Biological Chemistry*.

[76] Emmett M. J., Lim H. W., Jager J. (2017). Histone deacetylase 3 prepares brown adipose tissue for acute thermogenic challenge. *Nature*.

[77] Ferrari A., Galmozzi A., Mitro N. (2011). Inhibition of class I histone deacetylases unveils a mitochondrial signature and enhances lipid oxidation in skeletal muscle and adipose tissue. *Chemistry and Physics of Lipids*.

[78] Ferrari A., Longo R., Fiorino E. (2017). HDAC3 is a molecular brake of the metabolic switch supporting white adipose tissue browning. *Nature Communications*.

[79] Shares B. H., Busch M., White N., Shum L., Eliseev R. A. (2018). Active mitochondria support osteogenic differentiation by stimulating *β*-catenin acetylation. *Journal of Biological Chemistry*.

[80] Noer A., Lindeman L. C., Collas P. (2009). Histone H3 modifications associated with differentiation and long-term culture of mesenchymal adipose stem cells. *Stem Cells and Development*.

[81] Li Z., Liu C., Xie Z. (2011). Epigenetic dysregulation in mesenchymal stem cell aging and spontaneous differentiation. *PLoS One*.

[82] Tseng P. C., Hou S. M., Chen R. J. (2011). Resveratrol promotes osteogenesis of human mesenchymal stem cells by upregulating RUNX2 gene expression via the SIRT1/FOXO3A axis. *Journal of Bone and Mineral Research*.

[83] Fu Y., Zhang P., Ge J. (2014). Histone deacetylase 8 suppresses osteogenic differentiation of bone marrow stromal cells by inhibiting histone H3K9 acetylation and RUNX2 activity. *International Journal of Biochemistry & Cell Biology*.

[84] Schroeder T. M., Kahler R. A., Li X., Westendorf J. J. (2004). Histone deacetylase 3 interacts with runx2 to repress the osteocalcin promoter and regulate osteoblast differentiation. *Journal of Biological Chemistry*.

[85] Noce M., Mele L., Laino L. (2019). Cytoplasmic interactions between the glucocorticoid receptor and HDAC2 regulate osteocalcin expression in VPA-treated MSCs. *Cells*.

[86] Lee H. W., Suh J. H., Kim A. Y., Lee Y. S., Park S. Y., Kim J. B. (2006). Histone deacetylase 1-mediated histone modification regulates osteoblast differentiation. *Molecular Endocrinology*.

[87] Paino F., Noce M., Tirino V. (2014). Histone deacetylase inhibition with valproic acid downregulates osteocalcin gene expression in human dental pulp stem cells and osteoblasts: evidence for HDAC2 involvement. *Stem Cells*.

[88] Feigenson M., Shull L. C., Taylor E. L. (2017). Histone deacetylase 3 deletion in mesenchymal progenitor cells hinders long bone development. *Journal of Bone and Mineral Research*.

[89] Gao J., Feng Z., Wang X. (2018). SIRT3/SOD2 maintains osteoblast differentiation and bone formation by regulating mitochondrial stress. *Cell Death & Differentiation*.

[90] Lu D., Yao Y., Su Z. (2014). Downregulation of HDAC1 is involved in the cardiomyocyte differentiation from mesenchymal stem cells in a myocardial microenvironment. *PLoS One*.

[91] Yoon D. S., Choi Y., Jang Y. (2014). SIRT1 directly regulates SOX2 to maintain self-renewal and multipotency in bone marrow-derived mesenchymal stem cells. *Stem Cells*.

[92] Simic P., Zainabadi K., Bell E. (2013). SIRT1 regulates differentiation of mesenchymal stem cells by deacetylating *β*-catenin. *EMBO Molecular Medicine*.

[93] Buhrmann C., Busch F., Shayan P., Shakibaei M. (2014). Sirtuin-1 (SIRT1) is required for promoting chondrogenic differentiation of mesenchymal stem cells. *Journal of Biological Chemistry*.

[94] Dong X., Pan R., Zhang H., Yang C., Shao J., Xiang L. (2013). Modification of histone acetylation facilitates hepatic differentiation of human bone marrow mesenchymal stem cells. *PLoS One*.

[95] Liu J., Wang Y., Wu Y., Ni B., Liang Z. (2014). Sodium butyrate promotes the differentiation of rat bone marrow mesenchymal stem cells to smooth muscle cells through histone acetylation. *PLoS One*.

[96] Han B., Li J., Li Z. (2013). Trichostatin A stabilizes the expression of pluripotent genes in human mesenchymal stem cells during ex vivo expansion. *PLoS One*.

[97] Zych J., Stimamiglio M. A., Senegaglia A. C. (2013). The epigenetic modifiers 5-aza-2'-deoxycytidine and trichostatin A influence adipocyte differentiation in human mesenchymal stem cells. *Brazilian Journal of Medical and Biological Research*.

[98] Tan J., Huang H., Huang W. (2008). The genomic landscapes of histone H3-Lys9 modifications of gene promoter regions and expression profiles in human bone marrow mesenchymal stem cells. *Journal of Genetics and Genomics*.

[99] Tan J., Lu J., Huang W. (2009). Genome-wide analysis of histone H3 Lysine9 modifications in human mesenchymal stem cell osteogenic differentiation. *PLoS One*.

[100] Herlofsen S. R., Bryne J. C., Høiby T. (2013). Genome-wide map of quantified epigenetic changes during in vitro chondrogenic differentiation of primary human mesenchymal stem cells. *BMC Genomics*.

[101] Kronenberg H. M. (2003). Developmental regulation of the growth plate. *Nature*.

[102] Maruyama T., Mirando A. J., Deng C. X., Hsu W. (2010). The balance of WNT and FGF signaling influences mesenchymal stem cell fate during skeletal development. *Science Signaling*.

[103] Zhou S. (2011). TGF-*β* regulates *β*-catenin signaling and osteoblast differentiation in human mesenchymal stem cells. *Journal of Cellular Biochemistry*.

[104] Willert K., Brown J. D., Danenberg E. (2003). Wnt proteins are lipid-modified and can act as stem cell growth factors. *Nature*.

[105] Sekiya I., Vuoristo J. T., Larson B. L., Prockop D. J. (2002). In vitro cartilage formation by human adult stem cells from bone marrow stroma defines the sequence of cellular and molecular events during chondrogenesis. *Proceedings of the National Academy of Sciences of the United States of America*.

[106] He X. C., Zhang J., Tong W.-G. (2004). BMP signaling inhibits intestinal stem cell self-renewal through suppression of Wnt-*β*-catenin signaling. *Nature Genetics*.

[107] Jamora C., DasGupta R., Kocieniewski P., Fuchs E. (2003). Links between signal transduction, transcription and adhesion in epithelial bud development. *Nature*.

[108] Tu A. W., Luo K. (2007). Acetylation of Smad2 by the co-activator p300 regulates activin and transforming growth factor *β* response. *Journal of Biological Chemistry*.

[109] Kim K.-O., Sampson E. R., Maynard R. D. (2012). Ski inhibits TGF-*β*/phospho-Smad3 signaling and accelerates hypertrophic differentiation in chondrocytes. *Journal of Cellular Biochemistry*.

[110] Kim D. W., Lassar A. B. (2003). Smad-dependent recruitment of a histone deacetylase/Sin3A complex modulates the bone morphogenetic protein-dependent transcriptional repressor activity of Nkx3.2. *Molecular and Cellular Biology*.

[111] Jensen E. D., Schroeder T. M., Bailey J., Gopalakrishnan R., Westendorf J. J. (2009). Histone deacetylase 7 associates with Runx2 and represses its activity during osteoblast maturation in a deacetylation-independent manner. *Journal of Bone and Mineral Research*.

[112] Saidi N., Ghalavand M., Hashemzadeh M. S., Dorostkar R., Mohammadi H., Mahdian-shakib A. (2017). Dynamic changes of epigenetic signatures during chondrogenic and adipogenic differentiation of mesenchymal stem cells. *Biomedicine & Pharmacotherapy*.

[113] Tsuda M., Takahashi S., Takahashi Y., Asahara H. (2003). Transcriptional co-activators CREB-binding protein and p300 regulate chondrocyte-specific gene expression via association with Sox9. *Journal of Biological Chemistry*.

[114] Furumatsu T., Tsuda M., Yoshida K. (2005). Sox9 and p300 cooperatively regulate chromatin-mediated transcription. *Journal of Biological Chemistry*.

[115] Furumatsu T., Asahara H. (2010). Histone acetylation influences the activity of Sox9-related transcriptional complex. *Acta Medica Okayama*.

[116] Huang X., Xu J., Huang M. (2014). Histone deacetylase1 promotes TGF-*β*1-mediated early chondrogenesis through down-regulating canonical Wnt signaling. *Biochemical and Biophysical Research Communications*.

[117] Pei M., Chen D., Li J., Wei L. (2009). Histone deacetylase 4 promotes TGF-*β*1-induced synovium-derived stem cell chondrogenesis but inhibits chondrogenically differentiated stem cell hypertrophy. *Differentiation*.

[118] Bradley E. W., Carpio L. R., Olson E. N., Westendorf J. J. (2015). Histone deacetylase 7 (Hdac7) suppresses chondrocyte proliferation and *β*-catenin activity during endochondral ossification. *Journal of Biological Chemistry*.

[119] Fan Q., Tang T., Zhang X., Dai K. (2009). The role of CCAAT/enhancer binding protein (C/EBP)-alpha in osteogenesis of C3H10T1/2 cells induced by BMP-2. *Journal of Cellular and Molecular Medicine*.

[120] Zhao Q. H., Wang S. G., Liu S. X. (2013). PPAR*γ* forms a bridge between DNA methylation and histone acetylation at the C/EBP*α* gene promoter to regulate the balance between osteogenesis and adipogenesis of bone marrow stromal cells. *FEBS Journal*.

[121] Aoyama T., Okamoto T., Fukiage K. (2010). Histone modifiers, YY1 and p300, regulate the expression of cartilage-specific gene, chondromodulin-I, in mesenchymal stem cells. *Journal of Biological Chemistry*.

[122] Eid J. E., Garcia C. B. (2015). Reprogramming of mesenchymal stem cells by oncogenes. *Seminars in Cancer Biology*.

